# A Common Genomic Framework for a Diverse Assembly of Plasmids in the Symbiotic Nitrogen Fixing Bacteria

**DOI:** 10.1371/journal.pone.0002567

**Published:** 2008-07-02

**Authors:** Lisa C. Crossman, Santiago Castillo-Ramírez, Craig McAnnula, Luis Lozano, Georgios S. Vernikos, José L. Acosta, Zara F. Ghazoui, Ismael Hernández-González, Georgina Meakin, Alan W. Walker, Michael F. Hynes, J. Peter W. Young, J. Allan Downie, David Romero, Andrew W. B. Johnston, Guillermo Dávila, Julian Parkhill, Víctor González

**Affiliations:** 1 The Wellcome Trust Sanger Institute, Hinxton, Cambridge, United Kingdom; 2 Universidad Nacional Autónoma de México, Cuernavaca, México; 3 John Innes Centre, Norwich, United Kingdom; 4 Department of Biology, University of York, York, United Kingdom; 5 School of Biological Sciences, University of East Anglia, Norwich, United Kingdom; 6 Department of Biological Sciences, University of Calgary, Calgary, Canada; Wellcome Trust Centre for Human Genetics, United Kingdom

## Abstract

This work centres on the genomic comparisons of two closely-related nitrogen-fixing symbiotic bacteria, *Rhizobium leguminosarum* biovar *viciae* 3841 and *Rhizobium etli* CFN42. These strains maintain a stable genomic core that is also common to other rhizobia species plus a very variable and significant accessory component. The chromosomes are highly syntenic, whereas plasmids are related by fewer syntenic blocks and have mosaic structures. The pairs of plasmids p42f-pRL12, p42e-pRL11 and p42b-pRL9 as well large parts of p42c with pRL10 are shown to be similar, whereas the symbiotic plasmids (p42d and pRL10) are structurally unrelated and seem to follow distinct evolutionary paths. Even though purifying selection is acting on the whole genome, the accessory component is evolving more rapidly. This component is constituted largely for proteins for transport of diverse metabolites and elements of external origin. The present analysis allows us to conclude that a heterogeneous and quickly diversifying group of plasmids co-exists in a common genomic framework.

## Introduction

*Rhizobium etli* and *Rhizobium leguminosarum* bv *viciae* (henceforth called *R. leguminosarum*) are closely related species which are able to fix atmospheric nitrogen in symbiosis with specific leguminous plants. The common bean is the natural host of *R. etli* whereas *R. leguminosarum* interacts with peas, lentils, vetches and *Lathyrus* spp. Recently, we reported the complete genome sequences of a strain of *R. etli* and a strain of *R. leguminosarum* [1], [2], but no comprehensive genome comparison between these species had been carried out. To date, several other complete genome sequences of symbiotic nitrogen fixing bacteria have been published: *Mesorhizobium loti*, *Bradyrhizobium japonicum*, *B. spp. ORS278*, *B. spp. BTAi1* and *Sinorhizobium meliloti* [3]–[6]). Our comparisons of *R. etli* and *R. leguminosarum* show that: 1) *Rhizobium* genomes are composed of “core” and “accessory” components; 2) the chromosomes are markedly conserved in gene content (despite differences in size) and amongst the closest species gene order is also conserved; 3) the plasmids are heterogeneous in size and gene content and in some cases no synteny can be seen even in comparison with phylogenetic neighbours.

*Rhizobium* field isolates have the unusual feature of harbouring several plasmids, ranging in size from 100 kb to >1,000 kb and the plasmid profiles of a particular isolate can be used to type strains reliably [7]. Since *R. etli* CFN42 and *R. leguminosarum* 3841 are the most closely-related rhizobial species yet sequenced and both strains have six large plasmids, a detailed genome comparison between them may help us interpret the evolutionary history of these prototypical accessory elements. Indeed, whole genome comparisons allowed us to discern the distinctive properties of the core genome, and also to highlight the genetic differences between these species.

## Results

### Main features of the compared species

Both *R. etli* CFN42 and *R. leguminosarum* 3841 have large genomes composed of a circular chromosome and six large plasmids [1], [2]. The six CFN42 plasmids, pRetCFN42a-f, will be referred to as p42a-f throughout this article, whilst the six 3841 plasmids (sometimes known as pRL7JI-pRL12JI) are termed pRL7-12. The total size of the *R. etli* CFN42 genome is 1,221,081 bp shorter than that of *R. leguminosarum* 3841 (Table S1). The two smaller plasmids of *R. etli* are substantially larger than the two smallest plasmids of *R. leguminosarum*, whilst the opposite is the case for the other four plasmids (Table S1). *R. leguminosarum* plasmids comprise 34.8% of the total genome, whilst *R. etli* plasmids comprise an equivalent 32.9%. The two smallest *R. leguminosarum* plasmids are of lower than average GC content, whilst in *R. etli* the major nitrogen fixation plasmid (pSym; p42d) and the smallest plasmid (p42a) are the only plasmids of significantly lower GC content. The largest plasmids in both genomes resemble their corresponding chromosomes both in GC content and dinucleotide signatures. Symbiotic functions, specified by the *nod*, *nol*, *nif* and *fix* genes, are mainly encoded by a single plasmid (p42d in *R. etli* and pRL10 in *R. leguminosarum*), but other symbiosis-related genes are located on other plasmids and in the chromosome [1], [8]. The *R. etli* plasmid p42a is transferable at high frequencies and can help the mobilization of p42d [9]–[11] and p42d is also self-transmissible by conjugation [12] although its transfer ability is tightly repressed [13]. In *R. leguminosarum*, pRL7 and pRL8 are transmissible by conjugation, although neither carries a full set of *tra* genes [2].

### Phylogenomic relatedness between *R. etli* and *R. leguminosarum*

**Figure 1:**
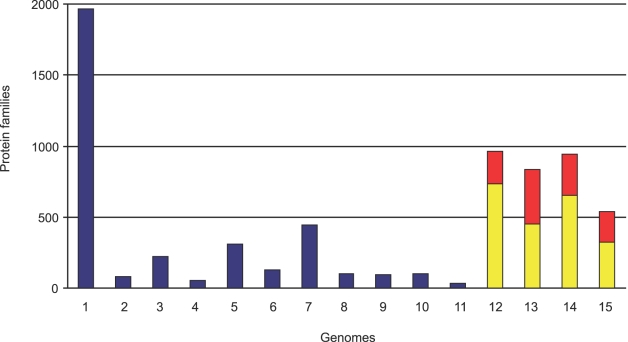
Distribution of protein families in the genomes of *S. meliloti* (S), *A. tumefaciens* (A), *R. leguminosarum* (L) and *R. etli* (E). - Bar number indicates the assignation of the protein families to the corresponding genome according to the following letters code: 1, SALE; 2, SAL; 3, ALE; 4, SAE; 5, SLE; 6. SA; 7, LE; 8, AL; 9, SE; 10, SL; 11, AE; 12, S; 13, A; 14, L; 15, E. Bars 12-15 show in red the proportion of orphan genes compared with those which match with known or hypothetical proteins present in the nr database of Genbank (yellow).

*R. leguminosarum* and *R. etli* are closely related species, judged by 16S rRNA comparisons and other molecular criteria (Figure S1). We first tested the consistency of these traditional phylogenies with genome phylogenies obtained with all individual proteins included in quartops (QUARtet of Orthologous Proteins). To do this, we incorporated two other species of the Rhizobiaceae family, *S. meliloti* and the non-nitrogen-fixing *Agrobacterium tumefaciens*, whose complete genomes are also available‥ A total of 33% and 39% of *R. leguminosarum* and *R. etli* proteins, respectively, were present in the Quartops; this equates to 2,392 predicted proteins representing core genes that are common to these four organisms (Table 1). Most of these predicted proteins are chromosomally encoded (2,054) but 338 belong to plasmids pRL9, pRL11 and pRL12. Three of the plasmids (pRL7, pRL8 and pRL10) do not have any proteins in Quartops. A total of 2,241 (85% of all proteins included in quartops) supports the phylogenetic relationship that proposes *R. leguminosarum* and *R. etli* are the most closely related. However, the high numbers of proteins absent from Quartops suggests that gene losses and gains might significantly have driven the diversification of the fast growing rhizobia. To investigate this area, we clustered all the predicted proteins of *R. etli*, *R. leguminosarum*, *S. meliloti* and *A. tumefaciens* into families by means of the MCL algorithm [14]. About 28% of the protein families identified (1,965 out 6,827) are shared by the four species, whereas about 10% (668) are only present in three species (Figure 1, bars 1–6). The rest of the protein families (13% or 908) occur in just two species. Most of these families (443) belong to the *R. etli-R. leguminosarum* pair, giving further support to the quartop phylogeny and the recent divergence of these two species (Figure 1, bars 7–11). Moreover, an appreciable number of families were particular to individual genomes. They belong to known and hypothetical families already present in the Genbank or they are orphan genes (Figure 1, bars 12–15). This confirms the previous findings that the coding potential of the rhizobial species is very variable while maintaining a stable common core.

**Table 1:** Quartops analysis with *R. leguminosarum*, *R. etli*, *A. tumefaciens and S. meliloti*.

	*Total proteins*	*No in quartops*	*Percentage in quartops*	*Rl-Re*	*Rl-At*	*Rl-Sm*
Chr	4736	2054	43.4	1951	25	23
pRL12	790	96	12.1	71	5	6
pRL11	635	147	23.1	136	-	5
pRL10	461	-	-	-	-	-
pRL9	313	95	30.3	83	4	4
pRL8	140	-	-	-	-	-
pRL7	188	-	-	-	-	-

### Genome synteny

**Figure 2:**
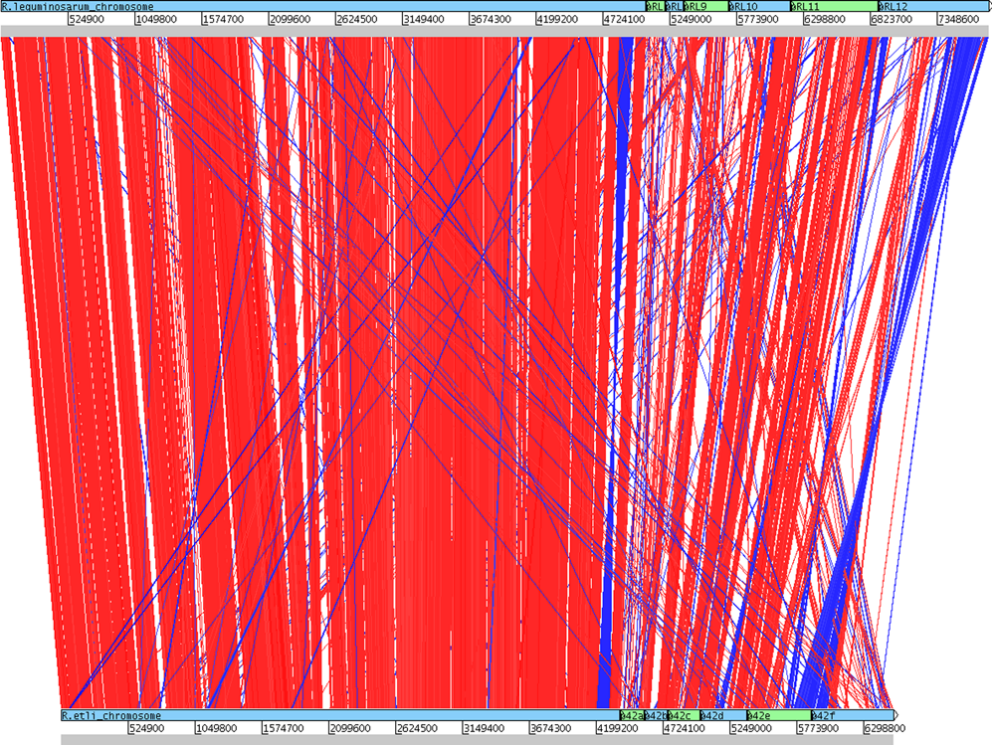
ACT View of Chromosome and Plasmids. The chromosomal and plasmid DNAs have been laid end-to-end and analysed using the Artemis comparison tool (ACT) [15]. Red bars represent close matches, whilst blue bars represent inverted close matches. The *R. leguminosarum* genome is at the top of the figure with replicons in the order Chromosome, pRL7, pRL8, pRL9, pRL10, pRL11, pRL12 whilst the *R. etli* genome is shown at the bottom of the figure in order Chromosome, p42a, p42b, p42c, p42d, p42e, p42f.

**Figure 3:**
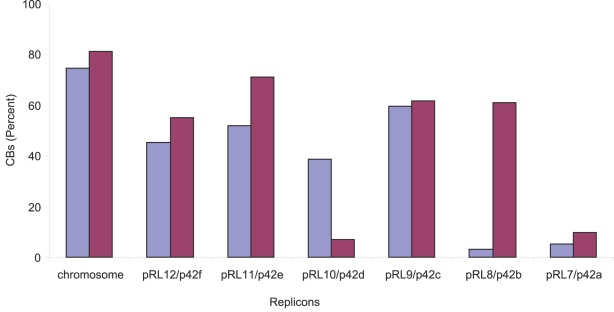
The proportion of synteny in the *R.etli* genome as compared to the *R. leguminosarum* genome. - The proportion of synteny is expressed as the percentage of the total DNA in CBs (Y axis) considering the total length of the pairs of replicons (X axis). Blue color *R. etli* CFN42; magenta, *R. leguminosarum* 3841.

To investigate whether the evolutionary relationship between *R. etli* and *R. leguminosarum* is also maintained at the level of gene order, the whole genomes were compared using ACT and Nucmer softwares [15], [16]. A clear syntenic pattern is distinguished between both chromosomes but it is also noticeable for some pairs of plasmids: (p42f-pRL12), (p42e-pRL11) and (p42b-pRL9) as well as large parts of p42c woth pRL10, suggesting a common origin (Figure 2). These observations are supported by the similarity of the replication genes, *repABC*, of those pairs of plasmids, as well as experimental demonstration of incompatibility between the plasmid pairs (Clark, Mattson, Garcia and Hynes, in preparation). Plasmids pRL7 and pRL8 appear to be unique to *R. leguminosarum* whilst p42a is peculiar to *R. etli* (see below). A more accurate measure of synteny between the genomes was obtained by calculating the length and number of colineal blocks (CBs). To do this, we employed a whole alignment obtained by Nucmer [16], then individual matches were clustered in CBs taking all the continuous segments separated by gaps less than 1kb. In total, 4,557,466 bp (70%) of the *R. etli* genome is contained in CBs with nucleotide identity about 85–95% (to *R. leguminosarum*). In the total genome of *R. leguminosarum*, 4,931,491 bp (63%) are contained in CBs. A total of 353 CBs >1 kb were recognized. The largest and most abundant (221) CBs are located on the chromosome and the rest on plasmids. Figure 3 shows that 81% and 74% of the chromosomes of *R. etli* and *R. leguminosarum* respectively are contained in CBs. Three of the *R. etli* plasmids have 44–58% of their genetic information in CBs that also occur in *R. leguminosarum*. Plasmids with fewer CBs are p42a, p42d, pRL7 and pRL8. Some of the plasmid pairs can be functionally identified by the presence of specific genes. For example, p42f and pRL12 carry some genes for flagellar biosynthesis (*flgLKE)* and for oxidative stress protection (*oxyR* and *katG*); p42e and pRL11 harbor cell division genes (*minCDE*), as well as thiamin, cobalamin, NAD biosynthetic genes (*thiMED*, *cobFGHIJKLM*, *nadABC*), and an isolated flagellin (*fla*) gene, as well as a rhamnose catabolism operon[17]. In some cases, *e.g. thiMED*, these genes are functionally interchangeable between these species [18]. A duplication of the *fixNOQP* operon in p42f [19], in *R. leguminosarum* is located in pRL9, a plasmid with homologous segments to p42b. Conjugative plasmids pRL8, pRL7 and p42a, which are otherwise unrelated to each other, have homologous *tra-trb* systems.

### Core genome composition and evolution

**Figure 4:**
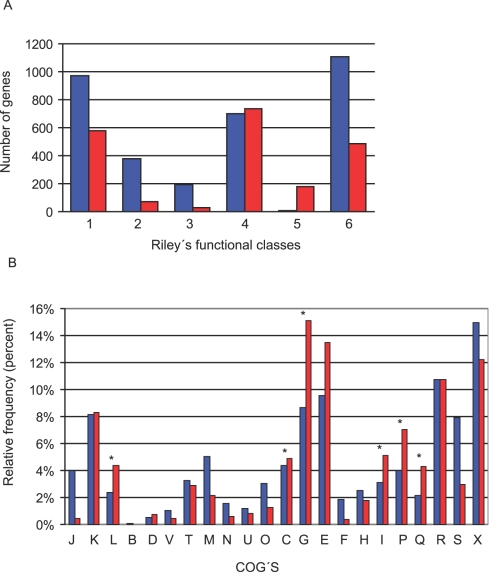
Functional bias in CHR-O (chromosomal only) and N-CHR (non-chromosomal) classes of homologues. - Figure 4a) Rileýs categories: 1. small molecule metabolism. 2. Macromolecule metabolism. 3. Structural elements. 4. Cell process. 5. External origin. 6. Miscellaneous. 4b) COGs functional classification. Bars indicate the relative frequency for each COG J, Translation, ribosomal structure and biogenesis; K, Transcription; L, Replication, recombination and repair; B, chromatin structure and dynamics; D, Cell cycle control; V, Defense mechanisms; T, Signal transduction mechanisms; M, Cell wall, membrane envelope biogenesis; N, Cell motility; U, Intracellular trafficking and secretion; 0, Postranslational modification and chaperones; C, Energy production and conversion; G, Carhohydrate transport and metabolism; E, Amino acid transport and metabolism; F, Nucleotide transport and metabolism; H, Coenzyme transport and metabolism; I, Inorganic ion transport and metabolism; P, inorganic ion transport and metabolism; Q, Secondary metabolites biosynthesis, transport and catabolism; R, General function prediction; S, function unknown; X, No COG.

*R. etli* and *R. leguminosarum* share 5,470 genes with approximately 89–100% similarity (see methods). A significant fraction of these common genes (3,359 or 62%) is solely present in both chromosomes (Chromosomal Only, CHR-O). The rest are situated either in the chromosome or plasmids or exclusively in the plasmids (Non-Chromosomal, N-CHR). Using the Riley classification scheme [20], CHR-O genes are overrepresented in the categories corresponding to small and macromolecule metabolism, structural elements, regulators and hypothetical conserved genes. In contrast, the N-CHR group tends to contain genes implicated in processes like chemotaxis, chaperones, transport, and elements of external origin (Figure 4a). A detailed classification using COGs [21] reveals other differences between CHR-O and N-CHR groups. Some of the COGs that are overrepresented in N-CHR are COG K (replication, recombination and repair) and the COGs related with predicted transport and metabolism of carbohydrates, amino acids, lipids and inorganic ions (COG G, E, I, and P) (Figure 4b), but not COGs related to information storage and processing.

**Figure 5:**
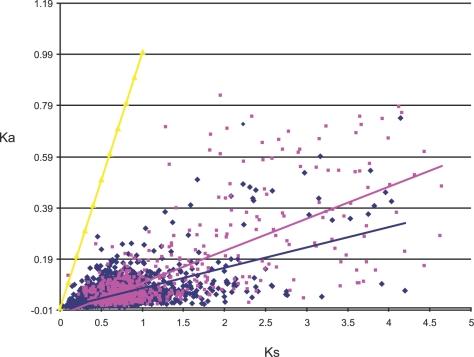
Rates of synonymous (Ks) and non-synonymous substitutions (Ka) in orthologous genes of *R. etli* and *R. leguminosarum*. - Neutrality line (Ka = Ks) is indicated in yellow. Linear regressions for CO class (blue color line and diamonds) and NC class (rose color line and diamonds) are indicated. As neutrality assumes equal nucleotide substitutions rates per synonymous and non-synonymous sites, points under the neutrality line indicate negative selection. Strong selective constraints are acting on genes of the CHR-O class (R2  =  0.6124; P≪0.001) but are slightly less intense for some genes of the N-CHR class (R2 =  0.5094), as can be seen by the dispersion of the rose color diamonds.

Differences between the CHR-O and the N-CHR gene compartments were also detected in regard to rates of evolution. To do this, we calculated the rates of nucleotide substitution per synonymous (Ks) and non-synonymous sites (Ka), for a subset of 2,917 single copy homologues (see methods; Figure 5). It is clear that both CHR-O and N-CHR homologous groups are under negative selection. Nevertheless, as seen by the slopes of the regression lines, the CHR-O group seems to be under stronger negative selection than the N-CHR group. However, many genes of the N-CHR group show higher Ka (>0.19) and Ks (>2.0) values than those of the CHR-O group. Therefore, negative selection is acting on the whole genome, but overall, the N-CHR gene compartment is less constrained.

### Mosaic replicons

**Figure 6:**
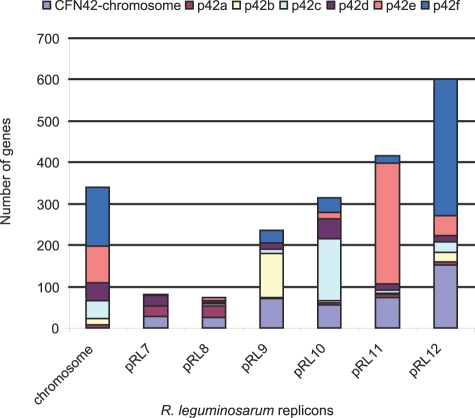
Composition of the *R. leguminosarum* and *R.etli* genomes according to N-CHR homologues. - The composition of the *R. leguminosarum* genome compared to the replicons of *R. etli* is shown. The replicon name is given at the base of the figure and color key to the right of the figure. Genes on the *R. etli* chromosome may be elsewhere on the *R. leguminosarum* genome (as shown in pale blue), genes from *R. etli* p42a (burgundy), p42b (cream), p42c (cyan), p42d (purple), p42e (salmon) and p42f are shown in royal blue.

Despite the high level of genome conservation, it is reasonable to expect that some degree of intra-genomic recombination has occurred since these two strains *R. etli* and *R. leguminosarum* had a common ancestor. This was substantiated by comparing the locations of the N-CHR group of genes in the different replicons of both genomes. Approximately 7% of the chromosomal genes of *R. leguminosarum* are represented in the plasmids of *R. etli*, and 10% of the chromosomal genes of *R. etli* are located in the *R. leguminosarum* plasmids. As shown before, some pairs of plasmids are likely equivalent in terms of their global similarity, but they are mosaic replicons that contain genes from the other replicons. For instance, pRL12 has significant similarity with p42f, but also possesses genes that in *R. etli* are chromosomal or on another plasmid (Figure 6). A similar pattern is observed in the other replicons (Figure 6). Such heterogeneous composition of the plasmids has precluded any attempt to make a reliable plasmid phylogeny. One way to assess the phylogenetic relatedness among plasmids is to compare their RepABC proteins that are essential components for plasmid replication [22]‥ However, we observed here that only the p42c-pRL10, p42d-pRL11 and p42f-pRL12 pairs carry closely related replication systems. They share nucleotide identities greater than 82% in the three proteins, whereas the RepABC proteins of the other plasmids are poorly related. Therefore, the replication genes might have been shuffled several times among the distinct plasmids, perhaps to allow a number of plasmids to coexist in the same cell.

### A potential symbiosis cassette

A comparison of the major symbiotic plasmids (pSyms) pRL10 and p42d shows that the *nif-nod* region in pRL10 is compacted into 60 kb, whereas in p42d it encompasses 125 kb. As many as 20 common *nod* and *nif* genes have been identified in comparisons among complete sequences of pSyms and symbiotic islands of different rhizobia [8]. The plasmid pRL10 contains 18 of these genes and has a particularly enhanced set of nodulation genes, including genes that lack homologs in *R. etli*, such as *nodTNMLEF* and *rhiABCR*. In contrast, pRL10 has a restricted set of genes for nitrogenase maturation, lacking *nifS*, *nifW*, *nifZ*, *nifX*, *iscN*, and *nifU*, which are present in *R. etli* and in other rhizobia. Besides the common nodulation genes, the *R. etli* pSym possesses *nolT*, *nolL*, *nolR*, *noeI*, *noeJ*, and a Type III secretion system.

**Figure 7:**
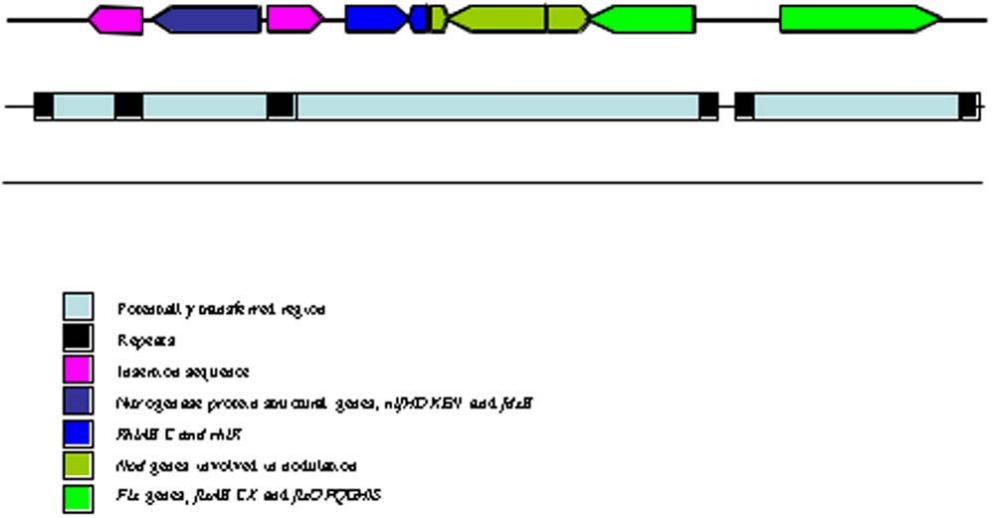
Diagram of the *R. leguminosarum* major nitrogen fixation gene cluster. - This cluster represents a potentially laterally transferred region of DNA. Major nitrogen fixation genes are represented as blocks and are as shown in the color key.

The symbiotic genes of *R. leguminosarum* may have been acquired by horizontal gene transfer, since an *in silico* analysis of pRL10 with the Alien Hunter program [23] reveals that its symbiotic gene cluster, which includes the *nif*, *nod*, *rhi* and *fix* genes, is located in a short potentially mobile region of DNA (∼63.5 kb). Internal to this region are the *nifNEKDH* genes that are found bounded by two identical IS element repeat regions. The *rhi* and *nod* gene cluster, together with *fixABCX*, lie adjacent on this potential genomic island and are potentially bounded by 20 bp repeats, whilst the *fixNOPQ* and *fixGHIS* genes lie immediately downstream on a separate putative genomic island of approximately 11,000 bp, potentially bounded by 18 bp repeats (Figure 7). It is possible that the *fixNOPQ*, *fixGHIS* island represents a second acquisition of DNA as an independent event. These adjacent symbiotic nitrogen fixation gene clusters are located in one particular region of the plasmid with six other short potentially horizontally transferred areas. The remainder of the pRL10 plasmid is highly similar to the p42c plasmid of *Rhizobium etli*. By contrast, the symbiotic nitrogen fixation genes are scattered throughout 125 kb of the p42d plasmid of *R. etli*. However, this region is surrounded by insertion sequences, which prompted the idea that it might be transposable [8]. When plasmid p42d was analysed by the Alien Hunter program 16 regions were detected as atypical. These regions contain the Type III transport system genes, *nod* genes, genes for virulence and conjugation (*vir* and *tra*), as well as cytochrome and chemotaxis genes (Figure S2). They are bordered by repeated sequences that might represent potential composite transposons when the repeats are homologous insertion sequences. Alternatively, the chimeric structure of p42d might have been the result of multiple gene exchanges and rearrangements.

### Physiological differences

The consequences of the evolutionary process of gain and losses are reflected in some physiological differences. For example, no candidate genes for respiratory nitrate reductases have been identified in the *R. etli* or *R. leguminosarum* genomes, however, the *nirK* gene for the respiratory nitrite reductase is present on *R. etli* p42f (RE1PF0000526). This gene appears to participate in nitrite detoxification [24]. Nitric oxide (NO) removal is encoded by *R. etli* as a predicted *norECBD* operon on the p42f plasmid located in proximity to the *nirKV* and probable regulators. These genes are absent in *R. leguminosarum*, although there are possible alternative NO consumption systems. One of such pathways encoded chromosomally by both *R. etli* and *R. leguminosarum* is *via* the assimilatory nitrite reductase. Another difference is the presence of erythritol catabolic genes, possibly originating from a horizontal transfer event, on pRL12 [25]. This gene cluster is absent from the CFN42 genome.

Since the lifestyles of *R. etli* and *R. leguminosarum* bv. *viciae* are similar, they may have similar responses to environmental stimuli. Thus, they may respond similarly with respect to environmental stimuli. For instance, population-density-dependent gene induction by N-acyl homoserine lactones (AHLs) influence symbiotic functions such as nodulation, nitrogen fixation, and surface polysaccharide production as well as several aspects of growth including plasmid transfer and stationary phase adaptation [8], [26] for reviews). Comparative analysis with known AHL regulators shows that there are 11 LuxR-type regulators in *R. etli* and 9 in *R. leguminosarum*. Some of them are known AHL regulators (CinR, TraR and RhiR) with associated AHL synthases (CinI, TraI, and RhiI) but there are also three other regulators, ExpR, AvhR and AsaR, for which there are no matching AHL synthases. In addition, we identified three LuxR-like sequences in *R. leguminosarum* (RL0606, RL0607 and RL3528) that matched the LuxR family over their entire length, but they could not be identified using protein domain searches. Two of these (RL0606 and RL0607) are highly conserved in *R. etli*, and in each case, they are located within a cluster of genes associated with bacterial motility, chemotaxis and flagella biosynthesis. Two related genes, *visN* and *visR* from *S. meliloti* strain RU10/406 act as global regulators of flagellar motility and chemotaxis, their products probably functioning as a heterodimer [27]. Although the third regulator (RL3258) also appears to be conserved in *R. etli* (CH03080) it has no known function. Remarkably, RhiR regulates the *rhiABC* operon that plays an undefined role in legume infection in *R. leguminosarum*, although this regulator is not present in *R. etli*, [28].

## Discussion

*Rhizobium* genomes consist of single circular chromosomes and several large plasmids. It is not understood why these genomes are so large and divided. Young *et al.* (2006) proposed that microbial life in the soil, a very heterogeneous environment, selects for a versatile genomes that encode multiple capabilities [2]. Therefore, genome comparisons between closely related *Rhizobium* species may indicate how variable these capabilities could be, as well as establishing whether they are distributed throughout the genome or in particular replicons. The comparative analysis presented here allows us to conclude that most of the differences between *R. etli* and *R. leguminosarum* tend to be in the plasmids. Previous genomic comparisons of *S. meliloti*, *A. tumefaciens*, and *R. etli* have shown that chromosomes are well conserved both in gene content and gene order, whereas plasmids have few common regions (*nif*-*nod*, *tra*-*trb*, *vir*, and others) and a lack of synteny [8]. These comparisons indicate that the plasmids in those three species are not closely related phylogenetically or that they have undergone many recombination events. Our analysis reveals many syntenic blocks exist between some pairs of plasmids of *R. etli* and *R. leguminosarum* (p42f-pRL12, p42e-pRL11 and p42b-pRL9 as well large parts of p42c with pRL10) suggesting a common origin. Plasmids of *R. etli* are smaller than those of *R. leguminosarum*, and 44–58% of their length is contained in CBs common to *R. leguminosarum*. Nonetheless, the phylogenetic relationships among the plasmids remain obscure.

A particular case of the mosaic structure of *Rhizobium* plasmids is shown by comparison of the symbiotic plasmids. In *R. leguminosarum* the pSyms are variable in size and also differ in *repC* group [2]. It has been noted that pRL10 and pRL1 (a pSym of 200 kb in *R. leguminosarum*) have a virtually identical *nod-nif* region, but the remainder of these plasmids appear to be dissimilar [2]. Speculatively, the entire symbiotic region may be a mobile element in *R. leguminosarum*, as has been proposed for the symbiotic region of p42d [8]. Although direct evidence for this scenario is still lacking, it is plausible given the observed recombinational plasticity displayed by rhizobial plasmids (reviewed by [29], [30]. Nevertheless, the overall structure of pRL10 more closely resembles p42c than p42d of *R. etli* (Figure 2). Extensive syntenic regions are common between pRL10 and p42c, accounting for 59% of the length of p42c (Figure 2). Thus, either pRL10 has gained a large insertion carrying the symbiotic nitrogen fixation functions, or p42c has suffered a large deletion of these genes. We show here that the former possibility could be plausible since the *nif-nod* region is a potential symbiotic cassette surrounded by repeated sequences. Furthermore, the structural differences between the pSyms of *R. etli* and *R. leguminosarum*, prompt us to suggest that they have evolved differently. In *R. leguminosarum* the Sym region resembles an specific “cassette”, whereas in *R. etli* the partial nucleotide sequence of different pSyms suggests that their diversification is driven by general recombination [31].

Some authors have proposed that bacterial genomes consist of “core” and “accessory” components [2], [32]. The “Core” component, exemplified by the chromosome, is more stable and changes more slowly over time than the “accessory” component. Plasmids are prototypical accessory elements composed of genes from different genomic contexts and evolutionary origin. As shown here, *R. etli* and *R. leguminosarum* are good models to study the evolution of plasmid (“accessory”) versus chromosome (“core”) evolution. Their chromosomes are nearly identical and harbor a distinct collection of plasmids that have evolved at different rates to the chromosome. It is tantalizing to speculate that these organisms can recruit plasmids from a pool in their soil environment [32]. Plasmids p42a, p42d, pRL7 and pRL8, in particular, seem to be the outliers. Other plasmids share many common regions and might have been part of the ancestral chromosome. Shuffling of the *repABC* genes might be a strategy to allow many plasmids to coexist in the same bacterium, and might explain the amazing plasmid diversity of *Rhizobium*. A more comprehensive picture of the evolution of the partitioned genomes can only be reached by comparing the respective plasmid pool of additional strains of *R. etli* and *R. leguminosarum* to describe how they are able to function in a common genomic framework.

## Methods

### Phylogenetic analysis

The 16S rRNA sequences were downloaded from EMBL for *R. leguminosarum* bv viciae Rlv3841, *R. etli* CFN42, *Agrobacterium tumefaciens* C58, *Sinorhizobium meliloti* 2011, *Mesorhizobium loti* MAFF303099, *Bradyrhizobium japonicum* USDA 110 and *Escherichia coli* T10. We first aligned the sequences using ClustalX [33] and generated a maximum likelihood tree using the PHYLIP package [34].

### Genome Comparisons

The complete nucleotide sequences of the *R. etli* CFN42 and *R. leguminosarum* Rlv3841 were obtained from Genbank (Accession numbers: *R. etli*, NC_007761-NC_007766, and NC_004041; *R. leguminosarum* NC_008378-NC008384). The sequences of the replicons for each genome were concatenated and used in a global comparison using ACT [15] and the Nucmer application of the Mummer package [16], with the default settings. To calculate the CBs, we took the nucmer.delta output and then parsed it with the show-coords utility. Syntenic segments >1 kb and separated by >1 kb were curated with *ad hoc* perl scripts and manual editing.

Clustering of protein families. First we did BLAST-P comparisons of “all versus all” complete proteomes of *R. etli*, *R. leguminosarum*, *S. meliloti* and *A. tumefaciens*. Clustering was achieved with MCL using an e-value of 10^−7^ and an inflation parameter of 1.5 [14].

### Homolog grouping and analysis of evolutionary rates

The most probable set of homologous proteins shared by *R. etli* and *R. leguminosarum* was identified using a reciprocal best-hit criterion. To that end, all *R. etli* predicted proteins were searched against the *R. leguminosarum* predicted proteome and *vice versa* using BLAST with cutoff e value of 10^−12^ and employing the Blosum-80 matrix [35]. In addition to this criterion, to be included in a homolog group the difference in length between the subject protein and query protein had to be <10%, the alignment region had to be at least of 80%, and there had to be a at least 50% similarity of both query and target sizes. We identified 5,470 homolog groups. The whole set was divided into two subdivisions. The first subdivision contains all the homolog groups in which there was only one protein per genome (unique bidirectional hits or possible orthologs, 2,917). The second subdivision contains homolog groups in which there is more than one protein in at least one genome, that is, possible paralogs (2,533). Further classification of the homolog groups was based on their localization. The “chromosomal-only” group (CHR-O) of homologs is present only in the chromosomes of both genomes, whereas the non-chromosomal group (N-CHR) was located either in chromosome or in plasmids, or exclusively in plasmids. Exclusive genes were recorded as those with no hits in the genomes at e-value of <10^−6^. The number of nucleotide substitutions per synonymous site “Ks” and the number of nucleotide substitutions per non-synonymous site “Ka” were determined with yn00 from PAML13.14 [36]

### Identification of genes involved in quorum sensing

We identified LuxI homologues using homology searches and independently determined proteins matching InterPro family IPR001690 (Autoinducer synthase). Both methods gave identical results. LuxR homologues were identified using homology searches as a guide, but were not by themselves used to identify likely LuxR proteins since the C-terminal DNA-binding domain in LuxR is also present at the C-terminus of a number of other proteins. Proteins containing the InterPro domain IPR005143 were identified, which corresponds to the N-terminal autoinducer-binding domain.

### Identification of horizontally acquired regions

Potentially horizontally acquired areas of DNA were identified with the Alien Hunter program, available from http://www.sanger.ac.uk/Software/analysis/alien_hunter.

## Supporting Information

Table S1 General features of the Genomes of *R.etli* and *R.leguminosarum*. A comparison of the main features of the genomes of *Rhizobium leguminosarum* and *Rhizobium etli*. Each replicon is described in terms of length in base pairs, %G+C content and number of coding sequences (CDS).
(0.04 MB DOC)

Figure S1 Phylogenetic tree. Maximum likelihood phylogenetic tree showing bacteria related to *R.etli* and *R.leguminosarum*
(0.08 MB TIF)

Figure S2 Chimeric structure of *R.etli* plasmid p42d. The circles show (outermost to innermost): 1. Atypical regions as bars of degraded colour (red to pale rose) according to the scores obtained from Alien Hunter (red, highest score 73 over a threshold of 32). 2. The 125 kb nif-nod region. 3, CDS of p42d according to the following colour code: blue, nodulation genes; yellow, nif genes; red, energy transfer genes (*fix* genes); green, insertion sequences; pink, transfer and replication genes; brown, hypotheticals; grey, transport (*vir* and *tss*III genes); sky blue, regulators. 4. Insertion sequences 5. Repeats from 100 to 300 identical nucleotides (black lines); repeats higher than 300 nucleotides (red lines).
(0.54 MB EPS)
