# Correction: A Common Genomic Framework for a Diverse Assembly of Plasmids in the Symbiotic Nitrogen Fixing Bacteria

**DOI:** 10.1371/annotation/4a58a9bd-7531-41d0-b101-53039a88c25b

**Published:** 2008-08-01

**Authors:** Lisa C. Crossman, Santiago Castillo-Ramírez, Craig McAnnula, Luis Lozano, Georgios S. Vernikos, José L. Acosta, Zara F. Ghazoui, Ismael Hernández-González, Georgina Meakin, Alan W. Walker, Michael F. Hynes, J. Peter W. Young, J. Allan Downie, David Romero, Andrew W. B. Johnston, Guillermo Dávila, Julian Parkhill, Víctor González

The resolution in Figure 7 is too low, making the figure illegible. Please view a revised version of Figure 7 here:

**Figure 7:**
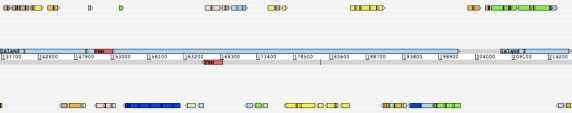
Diagram of the *R. leguminosarum* major nitrogen fixation gene cluster. This cluster represents a potentially laterally transferred region of DNA. Major nitrogen fixation genes and features are represented as blocks. The color key for the features shown is as follows: features labelled island_1 and island_2 show the two regions of potential horizontal gene transfer (light blue), nif structural genes for nitrogenase, nifDKHEN and fdxB, also nifAB (royal blue), fix genes, fixABCX and fixOPQGHIS (bright green gene cluster), gene products predicted to be membrane located (bright green, other), nod genes involved in nodulation (yellow gene cluster), genes involved in metabolism (yellow, other), rhiABC, rhiR and other potential regulators (light blue), repeats (dark pink), insertion sequences and transposon-derived (pink), pseudogenes and partials (brown), conserved hypotheticals (orange), hypothetical, no match (light green).

