# Supplementary material for: A Common Genomic Framework for a Diverse Assembly of Plasmids in the Symbiotic Nitrogen Fixing Bacteria
Source: PLoS One. 2008 Jul 2;3(7):e2567. doi: 10.1371/journal.pone.0002567 (PMC2434198; doi:10.1371/journal.pone.0002567)
Supplement: Table S1 — General features of the Genomes of R.etli and R.leguminosarum. A comparison of the main features of the genomes of Rhizobium leguminosarum and Rhizobium etli. Each replicon is described in terms of length in base pairs, %G+C content and number of coding sequences (CDS). (0.04 MB DOC) [file pone.0002567.s001.doc]

Supplementary Table 1.

| ***R.leguminosarum***  **Replicon** | **Base pairs** | **%G+C** | **CDS** | ***R. etli***  **Replicon** | **Base pairs** | **%G+C** | **CDS** |
| --- | --- | --- | --- | --- | --- | --- | --- |
| **Chromosome** | 5,057,142 | 61.1 | 4736 | **Chromosome** | 4,381,608 | 61.2 | 4067 |
| **pRL12** | 870,021 | 61.0 | 790 | **p42f** | 642,517 | 61.2 | 573 |
| **pRL11** | 684,202 | 61.0 | 635 | **p42e** | 505,334 | 61.7 | 459 |
| **pRL10** | 488,135 | 59.6 | 461 | **p42d** | 371,254 | 58.4 | 354 |
| **pRL9** | 352,782 | 61.0 | 313 | **p42c** | 250,948 | 61.5 | 234 |
| **pRL8** | 147,463 | 58.7 | 141 | **p42b** | 184,338 | 61.8 | 165 |
| **pRL7** | 151,546 | 57.6 | 189 | **p42a** | 194,229 | 58.0 | 182 |
| **Total** | 7,751,309 | 60.9 | 7265 | **Total** | 6,530,228 | 60.5 | 6034 |
