# Supplementary figures and images for: A Common Genomic Framework for a Diverse Assembly of Plasmids in the Symbiotic Nitrogen Fixing Bacteria
Source: PLoS One. 2008 Jul 2;3(7):e2567. doi: 10.1371/journal.pone.0002567 (PMC2434198; doi:10.1371/journal.pone.0002567)

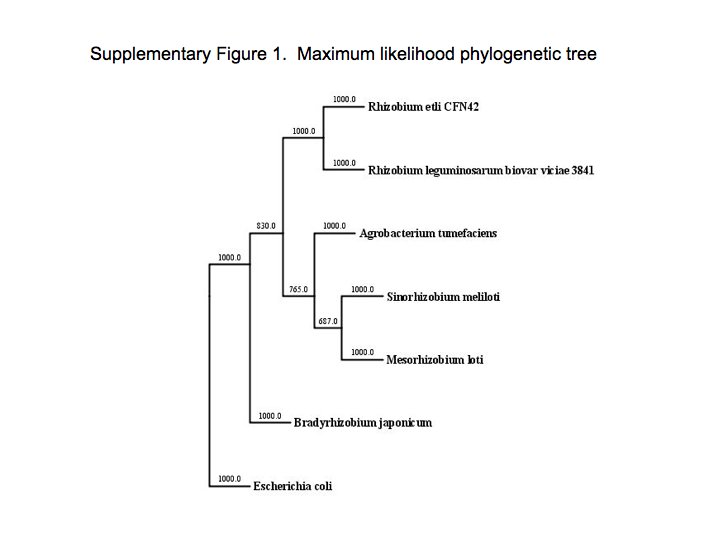

Supplement: Figure S1 — Phylogenetic tree. Maximum likelihood phylogenetic tree showing bacteria related to R.etli and R.leguminosarum (0.08 MB TIF) [file pone.0002567.s002.tif]
